# Supplementary material for: Rapid and assured genetic engineering methods applied to Acinetobacter baylyi ADP1 genome streamlining
Source: Nucleic Acids Res. 2020 Mar 30;48(8):4585–600. doi: 10.1093/nar/gkaa204 (PMC7192602; doi:10.1093/nar/gkaa204)
Supplement: gkaa204_Supplemental_Files [file gkaa204_supplemental_files.zip › Fig. S1 - PCR verification scheme with caption.pdf]

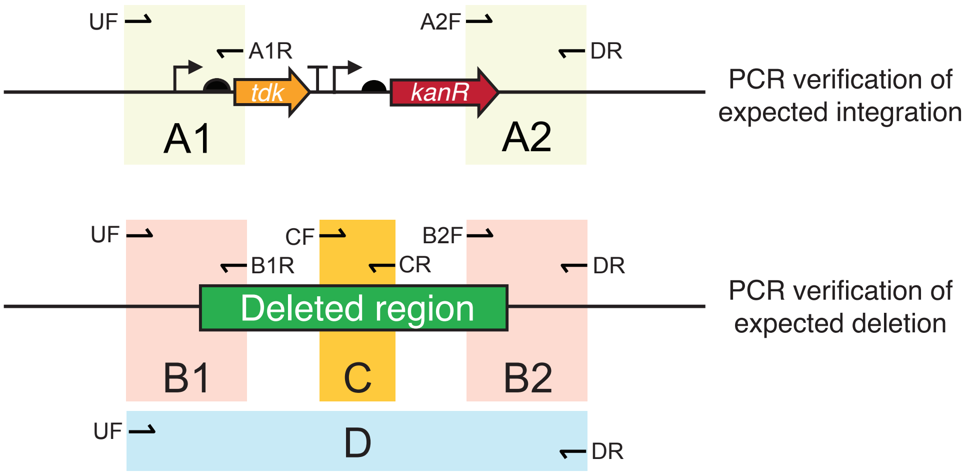


**Figure S1. PCR reactions used to validate multiple-gene deletion strain construction.** PCR reactions A1 and A2 check for insertion of the *tdk-kanR* cassette in the proper context. PCR reactions B1, B2, C, and D verify that the deleted region is missing from the edited chromosome. **Table S1** lists the primer sequences used in each reaction for each designed ADP1 deletion. **Table S3** reports the results of each type of PCR reaction for each candidate deletion strain.
